# Validation of the Chinese version of Emotion Regulation Strategies for Artistic Creative Activities Scale (ERS-ACA-C) among Chinese college students majoring in art and design

**DOI:** 10.3389/fpsyg.2025.1627567

**Published:** 2025-08-07

**Authors:** Aifang Yu, Zhidao Shi

**Affiliations:** ^1^Department of Public Art and Design, School of Fine Arts, Hangzhou Normal University, Hangzhou, China; ^2^Clinical Research Center for Mental Disorders, Shanghai Pudong New Area Mental Health Center, School of Medicine, Tongji University, Shanghai, China

**Keywords:** emotion regulation, emotion regulation strategies, artistic creative activities, college students, scale revision, reliability, validity

## Abstract

**Aim:**

Translating the Emotion Regulation Strategies for Artistic Creative Activities Scale (ERS-ACA) into Chinese and adapting it for Chinese college students majoring in art and design.

**Methods:**

Translated ERS-ACA into Chinese using a cross-cultural adaptation approach, and validated its psychometric properties among Chinese college students majoring in art and design. Three hundred and thirty-eight Chinese college students majoring in art and design were collected to test the scale’s reliability and validity, of which 150 students were retested 2 weeks later. Another 200 college students majoring in art and design were selected to perform Confirmatory Factor Analysis (CFA).

**Results:**

Item 1 of the Chinese version of ERS-ACA (ERS-ACA-C) was removed for its low correlation with the total score of ERS-ACA-C. We got the ERS-ACA-C with 17 items. Exploratory Factor Analysis (EFA) revealed that ERS-ACA-C has a general factor and three factors, consisting of 17 items. The explained variance of ERS-ACA-C was 61.098%. CFA validated that the three-factor model fit the data of ERS-ACA-C. The Cronbach’s alpha coefficient of ERS-ACA-C and its three factors was 0.883, 0.857, 0.875, and 0.854, respectively. Correlation analysis was performed between ERS-ACA-C and Self-Expression and Emotion Regulation in Art Making Scale (SERAMS) to calculate the criterion-related validity (*r* = 0.721, *p* < 0.001). The correlation coefficient r of the ERS-ACA-C’s test–retest reliability was 0.902 (*p* < 0.001). ERS-ACA-C’s split-half reliability was 0.754 (Spearman-Brown coefficient).

**Conclusion:**

ERS-ACA-C has relatively good reliability and validity. It can be used to assess the Emotion Regulation Strategies for Artistic Creative Activities of Chinese college students majoring in art and design in their artistic creative activities.

## 1 Introduction

Artistic creation activities have been widely recognized as effective tools for promoting mental health (Jean-Berluche, 2024). Artistic creation activities can make creators feel relaxed and enjoy themselves, helping them to learn new aspects of themselves and break free from constraints. For creators, this is an evolving process of initial struggle to later resolution, and about flow/losing themselves in the work, even lowering cortisol levels, a stress biomarker, in their saliva (Kaimal et al., 2016). By engaging in artistic creation activities, artists become more flexible in their thinking, cultivating their problem-solving abilities and psychological resilience, adaptability, and coping skills, thereby promoting their mental health (Kaufman, 2021; McDonnell, 2014).

However, research has found that art major college students, who dedicate most of their time to artistic creation, experience stress that significantly surpasses that of non-art majors (Kaufman, 2021; McDonnell, 2014). For example, financial stress is particularly acute for college students majoring in the arts. Many art schools are among the most expensive in higher education. A study in the United States found that the percentage with any debt and the amount of debt incurred have increased substantially among recent art graduates. Thirty-five percent of recent graduates said that debt levels significantly impacted their career decisions, compared to 14 % of students from other grades (Lena, 2014). In addition, due to long working hours, low income, and job instability in art-related professions after graduation (Oakley, 2009), as well as the prevailing view that arts careers are impractical and unprofitable, art major college students are also under pressure to make career choices (Lena, 2014). College students majoring in art also face significant academic pressure, requiring prolonged concentration with minimal rest during artistic creation (Brosamle et al., 2024). During the studio crit process, they had to present their ideas and/or artworks to the group, explain their creative thinking process, and receive formative feedback, mainly verbal, from the instructor or classmates, an experience many describe as terrifying, scary, or nerve-racking (Blair, 2007). They must create through iterative experimentation, failure, and revision (Phillips, 2019), a process that generates significant stress, fear, and anxiety (Anthony, 1991; Dannels and Martin, 2008).

Artistic creation highly values the originality of the work (Ellsworth, 2016). In the creative process, creators use improvisatory modes of thinking and action, and their final artworks often manifest high levels of indeterminacy (Smith, 2013), which is a severe challenge for all art major college students. Under the influence of practical pressures and cultural context, Chinese college students majoring in art may experience especially acute pressure to innovate (Li, 2024). To pass the National College Entrance Examination for Art Majors (Yikao), Chinese art students must focus on skill training, repetitively practicing, and imitating high-scoring artworks from Yikao (Fung Shung-Yu and Choi Yuet-Ngor, 2001). Furthermore, shaped by Confucian values and the artistic style of social realism from Russia, Chinese college art programs adopt a teacher-centered, skill-driven teaching model (Liang, 2019). This strict adherence to instructors’ guidance often limits students’ ability to express individual creativity and passion in their artwork (Liang, 2019). Over time, they may lack the courage, confidence, and imagination to express themselves creatively (Fung Shung-Yu and Choi Yuet-Ngor, 2001; Hong, 2020; Zhao et al., 2020), exacerbating stress and limiting creative output (Dou, 2015).

Research indicates that art major college students face intense pressure not experienced by students in many other majors, potentially leading to serious mental health issues such as anxiety and depression (Grant, 2010). Studies show they exhibit higher emotional exhaustion and depersonalization compared to other majors (Bernhard Ii, 2007). Repeated failures in the art creation process may foster learned helplessness (Putri Palupi, 2025), contributing to emotional disorders like anxiety, depression, and social withdrawal (Papworth et al., 2008). In the United States, approximately 25% of college art majors have used antidepressants, compared to 14% of the general population (Elias and Berg-Cross, 2009).

Artistic creation can regulate emotions for the general population, promoting psychological well-being (Fancourt and Ali, 2019; Wang et al., 2020; Zhang et al., 2023). However, for college students majoring in art and design, artistic creation to complete assignments or achieve academic success often increases psychological stress (Grant, 2010). Students experience heightened anxiety when facing assignments with ambiguous design objectives or time-intensive tasks that interfere with other coursework (Lee et al., 2024; Smith, 2013), and frustration when their artworks face harsh critique during studio crit (Blair, 2007; Buster and Crawford, 2010; Keeler Reeves, 2024). So far, the relationship between artistic creation and emotional regulation remains poorly understood, particularly for Chinese college students majoring in art and design (Hong, 2020). The emotional regulation strategies they adopt to manage significant creative pressure in their artwork creation are unclear, highlighting a research gap.

To address this gap, a tool is urgently needed to assess the emotion regulation strategies of Chinese college art and design majors during artistic creation. The Emotion Regulation Strategies for Artistic Creative Activities Scale (ERS-ACA) is a well-established research instrument developed by Fancourt et al. (2019) to assess emotion regulation strategies in artistic creation, with high reliability and validity. The absence of a Chinese version of the ERS-ACA currently limits our research progress. We translated the ERS-ACA into Chinese and tested its psychometric properties using a sample of Chinese college students majoring in art and design. This study provides a primary research tool for our subsequent research on emotion regulation in artistic creation among college students majoring in art and design in China.

## 2 Materials and methods

This study was conducted in accordance with the Declaration of Helsinki and was approved by the Fine Art School of Hangzhou Normal University (approval number 2024001). It was conducted from February 2024 to September 2024 in Hangzhou City, Zhejiang Province, China.

### 2.1 Participants

This study included undergraduate and graduate students majoring in art and design. Participants would be excluded from the study if they meet any of the following criteria:

- They are experiencing severe physical or mental illnesses that prevent them from completing the study.
- They refuse to participate in the study.

### 2.2 Instruments

#### 2.2.1 General information questionnaire

It includes several items related to the participants’ socio-demographic information, such as gender, age, and discipline.

#### 2.2.2 Emotion regulation strategies for artistic creative activities scale (ERS-ACA)

Fancourt developed the ERS-ACA to assess participants’ emotion regulation strategies during artistic creative activities. The theoretical basis for developing ERS-ACA originates from Gross’s process model of emotion regulation [emotion may be regulated at five points in the emotion generative process: (a) selection of the situation, (b) modification of the situation, (c) deployment of attention, (d) change of cognitions, and (e) modulation of responses] and integrates the dual process framework model of ERSs [ERSs are “explicit/conscious/voluntary”: including reappraisal, distraction, and suppression (Gyurak et al., 2011)], or “implicit/non-conscious/automatic”: including adaptation and the habitualisation of certain explicit ERSs as well as the self-perception theory of social cognitive psychology [individuals regulate their emotions through self-perception (self-esteem, self-efficacy, and agency)] (Alessandri et al., 2014; Braunstein et al., 2017). The scale consists of 18 items, including an overall “general” factor and three subscales: a 7-item “avoidance strategies” factor, a 6-item “approach strategies” factor, and a 5-item “self-development strategies” factor. All factors showed good internal reliability (Cronbach’s alpha: general factor = 0.93, factor 1 = 0.9, factor 2 = 0.88, and factor 3 = 0.88) (Fancourt et al., 2019). In this study, we would translate and adapt the ERS-ACA to make it suitable for assessing the emotion regulation strategies of Chinese college students majoring in art and design during their artistic creative activities.

#### 2.2.3 Self-expression and emotion regulation in art making scale (SERAMS)

We chose the Self-Expression and Emotion Regulation in Art Making Scale (SERAMS) to evaluate the criterion-related validity of ERS-ACA. The SERAMS is derived from the Chinese translation of the Self-expression and Emotion Regulation in Art Therapy Scale (SERATS). Haeyen developed SERATS as a validated tool to assess the effectiveness of art therapy for people with personality disorders (PD). Its theoretical foundation is based on Gross’s emotion regulation process model, as well as the integration of Personality Disorder Treatment Theory and Expressive Art Therapy Theory (Haeyen et al., 2018). PD patients often lack a sense of ownership due to problems with self, emotion, and behavior regulation. Through a period of art making in art therapy, they would learn to experience, become aware, and express feelings, as well as apply new behavioral patterns and gain self-insight to regulate emotions/feelings (letting out, making fall into place, or holding on to) and “reframing” their very often negative self-image (Haeyen and Noorthoorn, 2021). SERATS has nine items and one dimension, with responses formatted on a five-point Likert scale from 1 (never true) to 5 (almost always true). All nine items of the SERATS focus on the degree of successful self-expression, understanding, and regulation of emotions and behaviors that participants experience after art making (Haeyen et al., 2018). Both the ERS-ACA and SERATS are tools that assess emotion regulation in artistic creation, but they have some key differences. The ERS-ACA offers real-time quantification and mapping of how individuals regulate their emotions during the artistic process. It reveals how emotion regulation attempts unfold and identifies which strategies are employed, categorizing them into three dimensions: avoidance, approach, and self-development. In contrast, SERATS focuses on the progress individuals make in emotion regulation and self-expression following their participation in artistic activities. It evaluates whether artistic creation leads to adaptive insights and behavioral changes, thereby promoting the adaptive integration of personality. However, it is important to note that the items in SERATS do not capture the process perspective of avoidance strategies, which are included in the ERS-ACA.

Influenced by a variety of factors, Chinese art and design college students also face the problem of insufficient self-expression and emotion regulation in their art-making studies. Since there is no assessment tool in China, we translated SERATS into Chinese to quantitatively assess their changes in self-expression and emotion regulation abilities after art making training. Similar to SERATS, SERAMS also comprises nine items and a single dimension, demonstrating good psychometric properties, with a Cronbach’s alpha of 0.880 and a test–retest reliability correlation coefficient (r) of 0.889 (Yu and Shi, 2025).

### 2.3 Translation and adaptation of the scale

The developers of ERS-ACA allowed us to revise it through email communication. We utilized a cross-cultural adaptation approach to create a Chinese version of the ERS-ACA (Beaton et al., 2000; Wild et al., 2005). The steps for translation and adaptation are outlined below:

#### 2.3.1 Stage I: forward translation

A native English speaker and another native Chinese speaker translated ERS-ACA into Chinese.

#### 2.3.2 Stage II: synthesis of the translations

The research team members and the two translators mentioned above discussed the two translations and combined them into a common Chinese draft of ERS-ACA.

#### 2.3.3 Stage III: back translation

The common Chinese draft of ERS-ACA was back-translated into English by two other bilingual Chinese-English translators, resulting in two English back-translations of the ERS-ACA.

#### 2.3.4 Stage IV: expert committee review

Our research team members formed an expert committee with four translators (forward and backward translations), a linguist, and an epidemiologist to adapt the scale. They analyzed and compared the ERS-ACA, two forward translation versions, the common translation draft, and two backward translation versions, ultimately determining the initial Chinese ERS-ACA. The expert committee concluded that the initial Chinese ERS-ACA is equivalent to the original version of ERS-ACA in terms of semantics, idiomatic expressions, experience, and concepts.

#### 2.3.5 Stage VI: pretesting and cognitive interviews

Thirty college students who met the study criteria were recruited into a pretest and cognitive interview to check the comprehensibility of the initial Chinese ERS-ACA. The questionnaire filling process went smoothly. All participants reported no ambiguity about each item in the questionnaire.

#### 2.3.6 Stage VII: finalization of the Chinese version of ERS-ACA

After a discussion, the expert committee decided to accept the initial Chinese ERS-ACA as the final Chinese version of ERS-ACA (ERS-ACA-C).

### 2.4 Data collection

The researcher created the questionnaire using the Personal Computer Client of WJX software (provided by www.wjx.cn) and then generated the QR code for it. The QR code was then shared to a WeChat group of undergraduate or graduate classes in each major, and participants voluntarily filled out the questionnaire online after scanning the QR code with their cell phones. The questionnaire also included a question asking participants whether they would be willing to retest the questionnaire after 2 weeks. We randomly selected 150 people from those who agreed to participate in the retest. Two weeks after filling out the initial questionnaire, they would fill out the ERS-ACA-C online again.

### 2.5 Statistical analysis

Statistical analysis was conducted using SPSS 25.0. The socio-demographic information of the subjects was summarized using mean and standard deviation, frequency, and percentage. Continuous variables were presented as mean ± standard deviation (SD), while counts and percentages were used for categorical variables. A *p*-value of less than 0.05 indicates a statistically significant difference. AMOS 23.0 was utilized for the Confirmatory Factor Analysis (CFA).

#### 2.5.1 Item analysis

The homogeneity test was used to identify items with a low correlation with the total ERS-ACA-C score. If the Pearson correlation coefficient (r) between an item and the total ERS-ACA-C score is less than 0.4, the item would be removed (Nunnally and Bernstein, 1994).

#### 2.5.2 Validity analysis

ERS-ACA-C’s content validity, construct validity, and criterion-related validity were assessed.

Six senior teachers from the Department of Art and Design evaluated ERS-ACA-C’s item-level content validity index (I-CVI) and the scale-level content validity index (S-CVI). They were asked to rate the relevance of each item (1 = not relevant, 2 = somewhat relevant, 3 = quite relevant, 4 = highly relevant). The I-CVI of each item was computed as the number of teachers giving a rating of either 3 or 4, divided by the number of teachers—that is, the proportion in agreement about relevance. The scale-level content validity (S-CVI) is to compute the I-CVI for each item on the scale (the method is the same as above), and then calculate the average I-CVI across items (S-CVI/Ave). Retain items with ICV greater than or equal to 0.78. If the S-CVI/Ave is 0.9 or higher, content validity at the scale level is acceptable. If not, failing items should be deleted or modified and reassessed until they meet the criteria (Lynn, 1986; Polit et al., 2007).

We conducted Exploratory Factor Analysis (EFA) to examine the structural validity of ERS-ACA-C. The scale was considered suitable for factor analysis if the Kaiser-Meyer-Olkin measure of sampling adequacy (KMO) was ≥0.70 and Bartlett’s Test of Sphericity was statistically significant (*p* < 0.05) (Bartlett, 1950, 1951). Items with a Communalities below 0.2 (Spicer, 2005; Wu, 2010) and a Measure of Sampling Adequacy (MSA) less than 0.5 were deleted (Spicer, 2005). Principal Component Analysis (PCA) method combined with the Varimax Orthogonal Rotation method was used to analyze the data. Factors with Eigenvalues greater than one were retained (Kaiser and Rice, 1974). Each factor contained at least two items with loadings greater than 0.4(Samuels, 2017). Items with cross-loadings greater than 0.75 were deleted (Samuels, 2017). The scree test was used to verify the number of extracted factors visually.

The following indicators were used to evaluate the model’s goodness of fit in Confirmatory Factor Analysis (CFA). The standardized root mean square residual (SRMR) < 0.05; the root mean square error of approximation (RMSEA) < 0.08; The ratio of chi-square to degrees of freedom (CMIN/df) < 5; comparative fit index (CFI), goodness of fit index (GFI), and Tucker–Lewis index (TLI) value >0.9(Bentler, 1990; Hoyle, 1995).

Since the only standardized research tool currently available in China for assessing emotion regulation behaviors related to artistic creation among art and design college student is the SERAMS we previously translated, we conducted a Pearson correlation analysis between the ERS-ACA-C and the SERAMS to examine the criterion-related validity of the ERS-ACA-C. The criterion-related validity is acceptable if the Pearson correlation coefficient r ≥ 0.4 and is statistically significant (Kaplan and Saccuzzo, 2008).

#### 2.5.3 Reliability analysis

The acceptable standards for ERS-ACA-C’s reliability were as follows: Cronbach’s alpha ≥ 0.70. If the deletion of an item significantly increases Cronbach’s alpha value of ERS-ACA-C, that item should be removed (Nunnally and Bernstein, 1994). The Spearman-Brown coefficient (*ρ*) for split-half reliability should be ≥ 0.70. The intraclass correlation coefficient (ICC) should be ≥ 0.60 (Cicchetti and Sparrow, 1981). The Pearson correlation coefficient (r) for test–retest reliability should be ≥ 0.70 (Vilagut, 2014).

#### 2.5.4 Ceiling effect and floor effect

The items’ ceiling and floor effects were evaluated. When more than 15% of participants achieve the maximum or minimum score on a particular item, this suggests the presence of response bias within the data. Such a pattern reflects a ceiling or floor effect in the data (Terwee et al., 2007).

## 3 Results

Information was gathered from 538 individuals, with 338 of them randomly chosen to serve as subjects for the analysis of items, reliability, and validity. The other 200 individuals were utilized for Confirmatory Factor Analysis (CFA). Since participants submitted their responses only upon finishing the entire questionnaire, and all questions were required, there were no instances of missing data. Table 1 presents descriptive details regarding the characteristics of all participants.

**Table 1:** General characteristics of the participants in sample A and B.

Characteristics	Sample A	Sample B
N	338	200
Age (years)	21.28 ± 2.93	21.27 ± 2.96
Gender		
Male (%)	20.12%, (68/338)	23.50%, (47/200)
Female (%)	79.88%, (270/338)	76.50%, (153/200)
Sample size by grade		
Year 1 of bachelor	149	89
Year 2 of bachelor	101	51
Year 3 of Bachelor	28	19
Year 4 of bachelor	35	24
Year 1 of master	12	7
Year 2 of master	7	5
Year 3 of master	6	5
Sample size by major		
Environmental art design	44	36
Graphic design	42	35
Public art design	183	80
Fine art education	45	33
Painting	24	16
Total score of ERS-ACA-C	57.52 ± 10.70	58.01 ± 9.31
Total score of SERAMS	29.24 ± 8.48	

Data are means±SD or percentages. *N*, the sample size; Sample A, Participants for item, reliability, and validity analysis; Sample B, Participants for CFA; ERS-ACA-C, Chinese version of Emotion Regulation Strategies for Artistic Creative Activities Scale; SERAMS, Self-Expression and Emotion Regulation in Art Making Scale.

### 3.1 Item analysis

Pearson’s correlation coefficient between item 1 and the total score of the ERS-ACA-C was 0.233 < 0.4, indicating that item 1 was in poor homogeneity with the other 17 items and should be removed. The remaining 17 items passed the homogeneity test and were retained (Nunnally and Bernstein, 1994) (Table 2). Thus, we obtained an ERS-ACA-C with 17 items.

**Table 2:** Summary of ERS-ACA-C’s item analysis.

Items	Means ± SD	R	K/D
**1.** …I can block out any unwanted thoughts or feeling	3.01 ± 1.09	0.233^**^	Delete
**2.** …I can contemplate what is going on in my life with a clear mind	3.43 ± 1.01	0.588^**^	Keep
**3.** …I can shake off any anxieties in my life	3.33 ± 1.08	0.573^**^	Keep
**4.** …I feel I am in my own little bubble, away from ordinary worries	3.51 ± 1.10	0.607^**^	Keep
**5.** …I feel more confident in myself	3.26 ± 1.04	0.589^**^	Keep
**6.** …It boosts my self-esteem	3.38 ± 1.04	0.575^**^	Keep
**7.** …It gives me a sense of purpose	3.48 ± 1.00	0.541^**^	Keep
**8.** …It helps me forget about my worries	3.53 ± 1.10	0.625^**^	Keep
**9.** …It helps me refocus on what matter in my life	2.96 ± 1.08	0.619^**^	Keep
**10.** …It helps me to come to terms with my own emotions	3.45 ± 1.08	0.589^**^	Keep
**11.** …It helps me to disengage from things that are bothering me	3.24 ± 1.04	0.614^**^	Keep
**12.** …It helps me to put worries or problems I have in perspective	3.26 ± 1.06	0.596^**^	Keep
**13.** …It helps me to understand my own feelings on things that are on my mind	3.44 ± 1.04	0.577^**^	Keep
**14.** …It makes me feel detached from negative things in my life	3.31 ± 1.14	0.596^**^	Keep
**15.** …It makes me feel stronger in myself	3.52 ± 1.13	0.617^**^	Keep
**16.** …It makes me reflect on my emotions	3.56 ± 1.08	0.537^**^	Keep
**17.** …It reaffirms my identity	3.42 ± 1.02	0.534^**^	Keep
**18.** …It redirects my attention so I forget unwanted thoughts and feelings	3.43 ± 1.08	0.602^**^	Keep

**Correlation is significant at the 0.01 level; Question stems of ERS-ACA-C: As a college student majoring in Art and Design, you have participated in various artistic creation activities during your studies. Although people choose to engage in artistic creation for a variety of reasons, including simply to enjoy the process, to what extent do you agree with the following statements? When you participate in artistic creation activities. ERS-ACA-C, Chinese version of Emotion Regulation Strategies for Artistic Creative Activities Scale; SD, Standard deviation; R, Pearson correlation coefficient of each item to the total score of ERS-ACA; K/D, item was deleted or retained.

### 3.2 Validity analysis

#### 3.2.1 Content validity

Six senior teachers from the Department of Art and Design participated in the evaluation of the content validity of the ERS-ACA-C. The I-CVI of all items and the S-CVI/Ave of the ERS-ACA-C were both 1.0. They all met the criteria for judging a scale’s content validity (Lynn, 1986; Polit et al., 2007). The ERS-ACA-C has good content validity.

#### 3.2.2 Constructing validity

**Figure 1:**
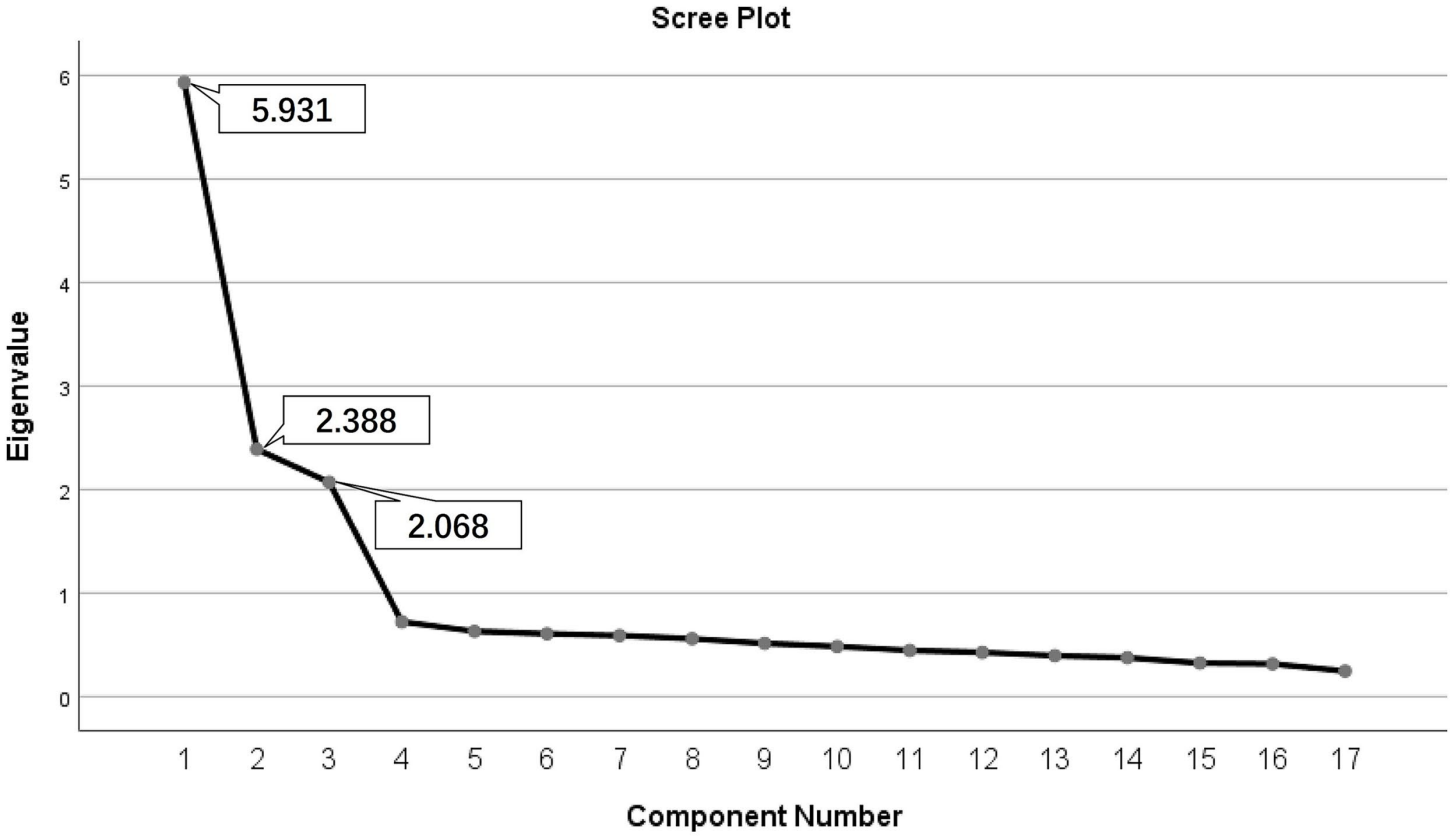
Scree plot. Three factors have a Kaiser’s eigenvalue ≥1; extraction method, principal component analysis.

Since the first item of the 18 items in ERA-ACA-C was deleted during item analysis, we conducted EFA on the remaining 17 items. Bartlett’s Test indicated that the 17-item ERS-ACA-C was suitable for factor analysis, with a KMO value of 0.884 ≥ 0.7. The chi-square value was 2547.873 (p<0.001) (Bartlett, 1950, 1951). The communalities of the items ranged from 0.571 to 0.683, all surpassing 0.2 (Wu, 2010; Spicer, 2005). The MSA values for the items ranged from 0.839 to 0.923, all exceeding 0.5 (Spicer, 2005), indicating that all items were suitable for the EFA. EFA was conducted using the PCA combined with the Varimax orthogonal rotation method. The Kaiser’s eigenvalue-over-one principle extracted three common factors, which explained 61.098% of the total variance. The initial eigenvalue was 5.931 (Table 3). The scree plot indicated that extracting three common factors was appropriate (Figure 1).

**Table 3:** Summary of exploratory factor analysis of ERS-ACA-C.

Items	MAS	IC	FLC1	FLC2	FLC3
Factor1. avoidance strategies					
1. …I can shake off any anxieties in my life	0.911	0.581	0.747		
2. …I feel I am in my own little bubble, away from ordinary worries	0.839	0.661	0.799		
3. …It helps me forget about my worries	0.888	0.631	0.767		
4. …It helps me to disengage from things that are bothering me	0.903	0.612	0.756		
5. …It makes me feel detached from negative things in my life	0.877	0.594	0.751		
6. …It redirects my attention so I forget unwanted thoughts and feelings	0.912	0.618	0.763		
Factor 2. approach strategies					
7. …I can contemplate what is going on in my life with a clear mind	0.923	0.571		0.729	
8. …It helps me refocus on what matter in my life	0.915	0.598		0.742	
9. …It helps me to come to terms with my own emotions	0.889	0.571		0.728	
10. …It helps me to put worries or problems I have in perspective	0.920	0.589		0.738	
11. …It helps me to understand my own feelings on things that are on my mind	0.868	0.615		0.768	
12. …It makes me reflect on my emotions	0.857	0.568		0.743	
Factor 3. self-development strategies					
13. …I feel more confident in myself	0.885	0.618			0.744
14. …It boosts my self-esteem	0.867	0.649			0.780
15. …It gives me a sense of purpose	0.861	0.633			0.780
16. …It makes me feel stronger in myself	0.863	0.683			0.787
17. …It reaffirms my identity	0.863	0.597			0.756
Eigenvalue			5.931	2.388	2.068
Variance explained (%)			21.793	20.704	18.601
Total variance explained (%)			61.098		

Extraction Method: Principal Component Analysis (Kaiser’s eigenvalue > 1); Rotation Method: Varimax with Kaiser Normalization; Three components extracted; Factor Loadings > 0.40 are reported. ERS-ACA-C, Chinese version of Emotion Regulation Strategies for Artistic Creative Activities Scale; MSA, Measures of Sampling Adequacy; IC, item’s communalities; FLC1, factor loadings of component 1; FLC2, factor loadings of component 2; FLC3, factor loadings of component 3.

Factor 1 of 17-item ERS-ACA-C contains six items, items 1, 2, 3, 4, 5, and 6. These six items all belong to the avoidance strategies factor of the original ERS-ACA. Therefore, factor 1 was also named avoidance strategies. Factor 2 of 17-item ERS-ACA-C contains six items, items 7, 8, 9, 10, 11, and 12. These six items all belong to the approach strategies factor of the original ERS-ACA. Therefore, factor 2 was also named approach strategies. Factor 3 of 17-item ERS-ACA-C contains five items: 13, 14, 15, 16, and 17. These five items all belong to the self-development strategies factor of the original ERS-ACA. Therefore, factor 3 was also named self-development strategies (Fancourt et al., 2019). EFA also extracted a general factor that included all 17 items. Factor loadings ranged from 0.534 to 0.631.

**Figure 2:**
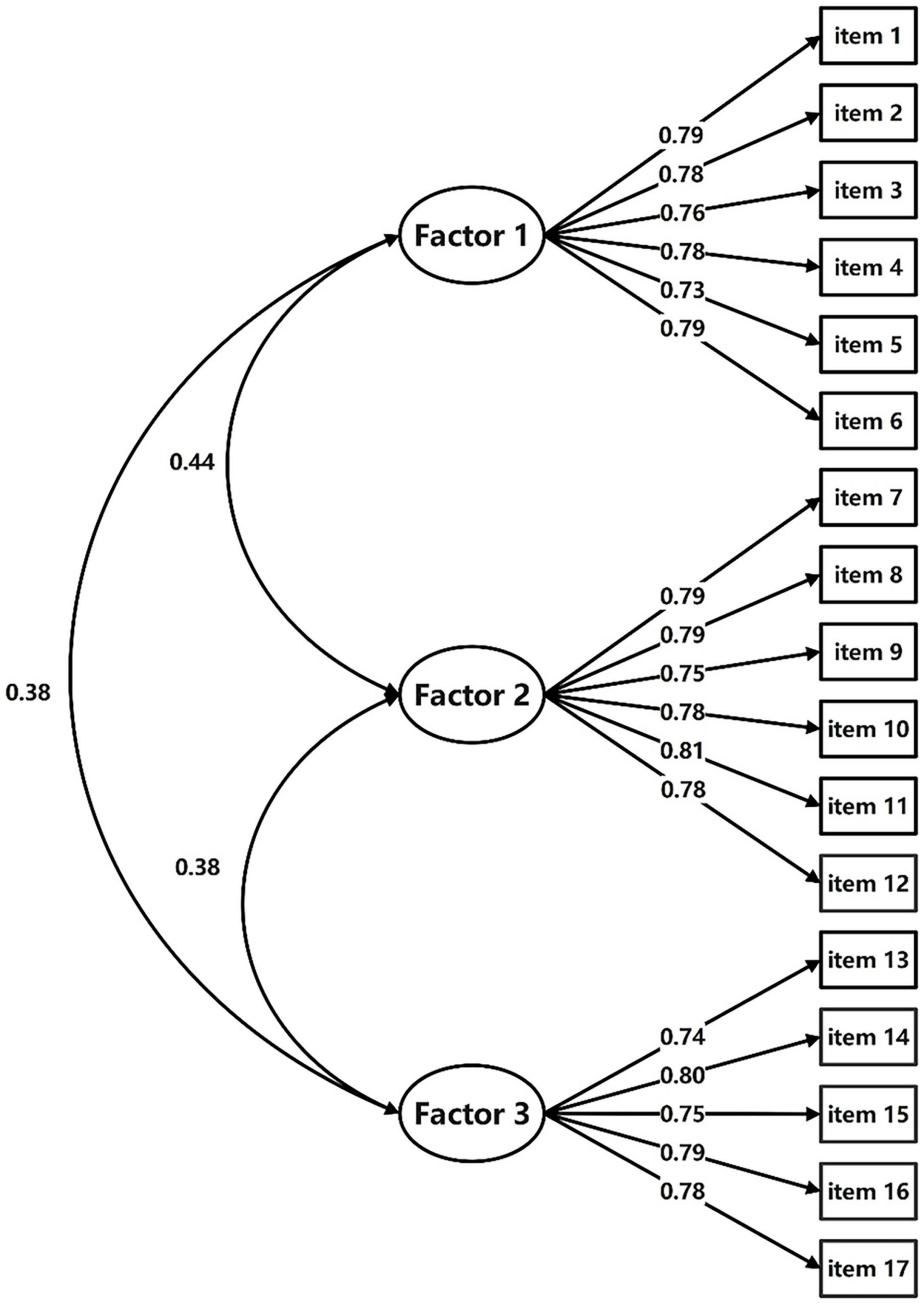
Path diagram for ERS-ACA-C’s first-order CFA. Notes: ERS-ACA-C, Chinese version of Emotion Regulation Strategies for Artistic Creative Activities Scale; CFA, confirmatory factor analysis; Factor 1, avoidance strategies factor; Factor 2, approach strategies factor; Factor 3, self-development strategies factor.

**Figure 3:**
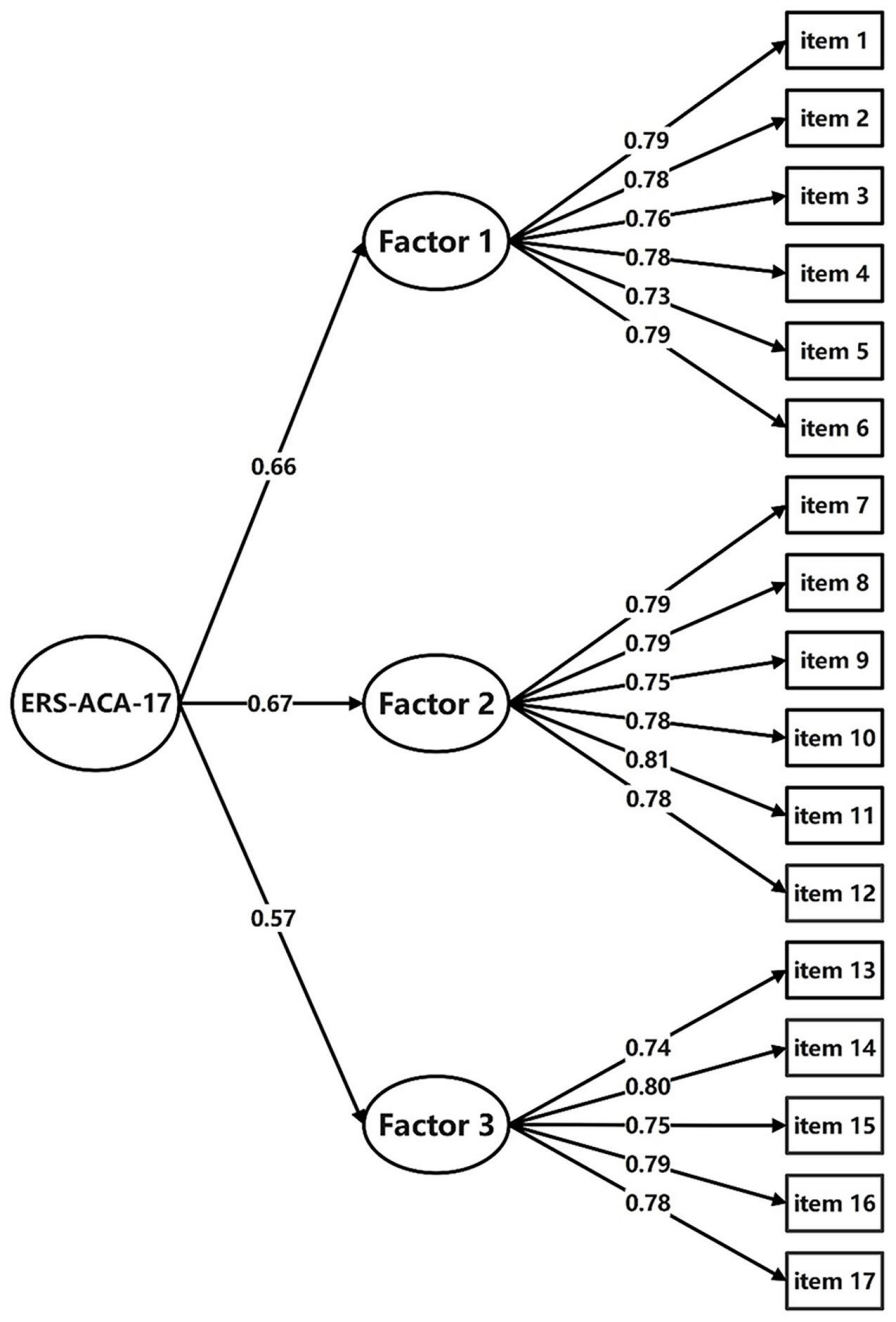
Path diagram for ERS-ACA-C’s second-order CFA. ERS-ACA-C, Chinese version of Emotion Regulation Strategies for Artistic Creative Activities Scale; CFA, confirmatory factor analysis; Factor 1, avoidance strategies factor; Factor 2, approach strategies factor; Factor 3, self-development strategies factor.

Results of the CFA confirmed the structure of the 17-item ERS-ACA-C with a good model fit with CMIN/DF = 1.091 < 5; GFI = 0.925, CFI = 0.947, TLI = 0.938, their values all >0.9; the SRMR = 0.0496 < 0.05, and the RMSEA = 0.021 < 0.05. Standardized factor loadings ranged between 0.727 and 0.811. So, ERS-ACA-C’s first and second-order model structures are appropriate (Figures 2, 3).

#### 3.2.3 Criterion-related validity

The total score of ERS-ACA-C and its three factor scores are positively correlated with the total score of SERAMS; The Pearson correlation coefficients were 0.721, 0.556, 0.559, and 0.518, respectively (*p* < 0.001). The criterion-related validity of ERS-ACA-C and its three factors was acceptable (Kaplan and Saccuzzo, 2008).

### 3.3 Reliability analysis

The Cronbach’s alpha coefficient of ERS-ACA-C was 0.883 (p<0.001). Removing an item from ERS-ACA-C would not increase its Cronbach’s alpha value. All seventeen items in the ERS-ACA-C were retained. The Cronbach’s alpha coefficient of factor 1, factor 2, and factor 3 was 0.857, 0.875, and 0.854, respectively. The Cronbach’s alpha of ERS-ACA-C and its three factors is appropriate (DeVellis, 2016). ICC of ERS-ACA-C and its three factors was 0.883 (95% CI 0.864–0.900), 0.857 (95% CI 0.832–0.880), 0.875 (95% CI 0.853–0.894), 0.854 (95% CI 0.828–0.878) respectively, which are all greater than 0.75 and met Cicchetti’s criteria for good (Cicchetti and Sparrow, 1981). The Spearman-Brown coefficient values of ERS-ACA-C and its three factors were 0.754, 0.869, 0.866, and 0.853, respectively, which are acceptable (DeVellis, 2016). Two weeks later, we used ERS-ACA-C to measure 150 subjects again. The Pearson correlation coefficient r of the test–retest reliability of ERS-ACA-C and its three factors was 0.902, 0.861, 0.896, and 0.865, respectively (*p* < 0.001). The test–retest reliability of ERS-ACA-C and its three factors is acceptable (Vilagut, 2014) (Table 4).

**Table 4:** Summary of ERS-ACA-C’s reliability.

Item	α1	α2
Factor 1. avoidance strategies		
1. …I can shake off any anxieties in my life	0.877	0.835
2. …I feel I am in my own little bubble, away from ordinary worries	0.876	0.831
3. …It helps me forget about my worries	0.877	0.834
4. …It helps me to disengage from things that are bothering me	0.876	0.832
5. …It makes me feel detached from negative things in my life	0.877	0.830
6. …It redirects my attention so I forget unwanted thoughts and feelings	0.879	0.838
Factor 2. approach strategies		
7. …I can contemplate what is going on in my life with a clear mind	0.877	0.858
8. …It helps me refocus on what matter in my life	0.876	0.848
9. …It helps me to come to terms with my own emotions	0.875	0.851
10. …It helps me to put worries or problems I have in perspective	0.876	0.854
11. …It helps me to understand my own feelings on things that are on my mind	0.877	0.857
12. …It makes me reflect on my emotions	0.876	0.853
Factor 3. self-development strategies		
13. …I feel more confident in myself	0.876	0.827
14. …It boosts my self-esteem	0.877	0.821
15. …It gives me a sense of purpose	0.878	0.826
16. …It makes me feel stronger in myself	0.875	0.815
17. …It reaffirms my identity	0.879	0.832

ERS-ACA-C, Chinese version of Emotion Regulation Strategies for Artistic Creative Activities Scale; α1, ERS-ACA-C’s Cronbach’s Alpha if item deleted; α2, factor’s Cronbach’s Alpha if item deleted; SHR: split-half reliability; TRR: test–retest reliability. Reliability of Factor 1: Cronbach’s α = 0.857; ICC: 0.857 (95% CI 0.832–0.880); SHR: *ρ* = 0.869; TRR: *r* = 0.861. Reliability of Factor 2: Cronbach’s α = 0.875; ICC: 0.875 (95% CI 0.853–0.894); SHR: ρ = 0.866; TRR: *r* = 0.896. Reliability of Factor 3: Cronbach’s α = 0.854; ICC: 0.854 (95% CI 0.828–0.878); SHR: *ρ* = 0.853; TRR: *r* = 0.865. Reliability of ERS-ACA-C: Cronbach’s *α* = 0.883; ICC: 0.883 (95% CI 0.864–0.900); SHR: *ρ* = 0.754; TRR: *r* = 0.902.

### 3.4 Ceiling effect and floor effect

No participants achieved the lowest total score of 17 or the highest total score of 85. Therefore, no subject response bias was observed in the current study (Terwee et al., 2007). There is no ceiling effect or floor effect in the data.

## 4 Discussion

Under the influence of real-life pressures and cultural background, college students majoring in art and design in China suffer from significant deficiencies in self-confidence and creative expression. This results in pronounced stress related to innovation during their artistic creation, which in turn triggers a series of emotional adjustment issues and academic problems (Fung Shung-Yu and Choi Yuet-Ngor, 2001; Hong, 2020; Li, 2024; Zhao et al., 2020). As faculty members in art and design, although we have already observed numerous emotional responses exhibited by these college students during the process of artistic creation, we still lack clarity—due to the absence of appropriate assessment tools—on exactly what kinds of emotion regulation strategies these students adopt in order to cope with their creative stress, to facilitate the orderly progression of their artistic creation process.

Fancourt et al. (2019) developed the ERS-ACA to assess the types of emotion regulation strategies employed by creators during artistic creative activities, using a general population sample in the United Kingdom. The ERS-ACA categorizes emotion regulation strategies in artistic creation into three broad types: avoidance, approach, and self-development strategies. It demonstrates sound psychometric properties, with Cronbach’s alpha values of 0.93 (total factor), 0.90 (factor 1), 0.88 (factor 2), and 0.88 (factor 3). As an effective and practical research tool, the ERS-ACA has deepened our understanding of the mechanisms by which artistic creative activities lead to emotional engagement and processing. To address the practical challenges we face in teaching, we translated the ERS-ACA into Chinese and conducted a psychometric evaluation of the translated version.

The original English version of the ERS-ACA consists of a 18-item scale comprising an overall ‘general’ factor of ERSs alongside three subscales: a 7-item factor comprising ‘avoidance strategies’ (such as distraction, suppression and detachment), a 6-item factor comprising ‘approach strategies’ (such as acceptance, reappraisal and problem solving), and a 5-item factor comprising ‘self-development strategies’ (such as enhanced self-identify, improved self-esteem and increased agency) (Fancourt et al., 2019) (Table 2).

CFA revealed that the overall scale structure of the ERS-ACA-C exhibits a high degree of similarity to that of the original English version of the ERS-ACA. The number and content of items within the “approach strategies” and “self-development strategies” factors are exactly the same between the ERS-ACA-C and the ERS-ACA. The only difference lies in the “avoidance strategies” factor, which in the ERS-ACA-C lacks Item 1 (When participate in artistic creation activities, I can block out any unwanted thoughts or feeling) that is present in the ERS-ACA. As a result, the general factor of “Emotion Regulation Strategies” (ERSs) in the ERS-ACA-C contains 17 items (while the original ERS-ACA includes 18), and the “avoidance strategies” factor consists of 6 items (compared to 7 in the original). The deletion of Item 1 was due to its low item-total correlation with the overall score of the ERS-ACA-C during item analysis (*r* = 0.233), which warrants further discussion.

One notable perspective is that the point in the emotion regulation process targeted by Item 1 differs from those targeted by the other items. Research in psychology has found that the impact of emotions on creativity is not fixed or singular—different types of emotions may each play a uniquely positive role depending on the task context. Both positive and negative emotions can promote creativity (Baas et al., 2008). For example, when a creative task is framed as entertainment and enjoyment, participants in a positive emotional state tend to be more creative than those in a negative emotional state; however, when the task is framed as serious or performance-related, participants in a negative emotional state often exhibit greater creativity (Baas et al., 2008). When a person is deeply engulfed in intense negative emotions that evoke feelings of avoidance, fear, or anxiety, their creativity is often inhibited, making it difficult for new ideas to emerge. Research findings in the field of art psychology has found that for artistic creators, entering a state of “flow” (that is, deep emotional immersion and optimal cognitive concentration) is crucial for producing high-quality artworks (Csikszentmihalyi, 1990). However, even when artistic creators are fully absorbed in the task at hand and in a state of “flow,” automatic and unconscious information processing still occurs and can influence behavior (Dehaene et al., 2006; Van Gaal et al., 2008). At such times, the influence of the subconscious may be diminished but remains active rather than completely absent (Csikszentmihalyi, 1990). Therefore, the claim made in Item 1—that one can block out any unwanted thoughts or feeling—is, in practice, unattainable.

Gross’s emotion regulation process model asserts that emotions can be regulated at five stages in the emotion generation process: (a) situation selection, (b) situation modification, (c) attentional deployment, (d) cognitive change, and (e) response modulation. According to this model, emotion regulation efforts may target two different points in the emotion generation process. Antecedent-focused emotion regulation occurs at the front end or very early stage of the emotion generation process, whereas response-focused emotion regulation takes place at the later stage, or after the emotional response tendencies have been triggered. Thus, antecedent-focused regulation prevents emotions from overflowing at the outset, while response-focused regulation serves to “finish off” or dampen the emotion (Gross, 1998). Item 1, “I can block out any unwanted thoughts or emotions,” is a response-focused emotion regulation process, specifically representing an inhibition of emotional behavior. The term “block out” in Item 1 suggests that respondents can completely inhibit or sever the unwanted emotional content, representing a later, response-oriented regulatory approach, namely expressive suppression. Whether under high-emotion or low-emotion conditions, expressive suppression requires continuous monitoring of ongoing expressive behaviors, which occupies working memory, increases physiological arousal, and incurs significant cognitive cost (Richards and Gross, 2000). In contrast, the other items in ERS-ACA, —such as “redirects my attention,” “detached from negative things,” “refocus on what matters in my life,” “to come to terms with my own emotions,” “put worries or problems I have in perspective,” “understand my own feelings on things that are on my mind,” “feel more confident in myself,” “feel stronger in myself,” etc.—are each part of the emotion regulation processes such as Attentional Deployment and Cognitive Change, belonging to the antecedent-focused emotion regulation in Gross’s emotion regulation process model, and thus incur relatively lower cognitive costs (Richards and Gross, 2000). Because Item 1 and the rest of the items pertain to emotion regulation efforts at two different moments in the emotion generation process, from this perspective, Item 1 in the original ERS-ACA is heterogeneous relative to the other items.

At the same time, the differing levels of acceptance of Item 1 across cultural contexts may offer another explanation for its inclusion in one version and exclusion in the other. Cross-cultural research indicates that by adopting culturally different emotion regulation strategies, individuals actively shape their emotional experiences, thereby playing a positive role in maintaining and sustaining cultural scripts (Miyamoto and Ma, 2011). Western cultures place great emphasis on open and authentic emotional expression, considering it the core of individual identity. In these societies, both positive and negative emotions should be expressed clearly and energetically, to reflect honesty and psychological well-being (Markus and Kitayama, 1991). By contrast, the dominant cultural script in Eastern cultures not only seeks the Doctrine of the Mean through balancing the experience of positive and negative emotions, but also integrates a dialectical approach that accepts the possibility of the coexistence of positive and negative emotions (Miyamoto and Ma, 2011). Their cultural norms place greater emphasis on collectivism, stress the maintenance of social harmony and the protection of face, and therefore value restraint and moderation in emotional expression; excessively intense emotional expressions are generally not encouraged, as they may be seen as behaviors that undermine interpersonal relationships and group cohesion.

In Eastern cultures, individuals are socialized to suppress or tone down emotional expression in order to meet contextual demands and contribute to the maintenance of social order (Markus and Kitayama, 1991; Matsumoto, 1990). Accordingly, when completing the ERS-ACA scale, Chinese art students may be less inclined to endorse Item 1 (“I can block out any unwanted thoughts or emotions”), as its absolutist wording conflicts with their cultural tendency to value social harmony and collective ideals, and to favor emotional restraint and balance. By contrast, the participants involved in the development of the original ERS-ACA were predominantly White British individuals who identify with Western cultural norms, which tend to regard open and direct emotional expression as an important reflection of personal authenticity. As a result, they are generally more likely to accept such absolutist phrasing. Therefore, Item 1 was retained in the ERS-ACA but removed from the ERS-ACA-C.

Additionally, from a psychometric perspective, overly absolute item wording often leads respondents to provide extreme or inflexible answers. This, in turn, decreases response variability and weakens the item’s discriminative power as well as its correlation with the total scale score (DeVellis, 2016; Zumbo, 1999). Moreover, such absolute phrasing typically fails to capture the subtle and continuous nature of individuals’ emotional regulation experiences. As a result, the item tends to correlate less with the overall construct, thereby compromising internal consistency and construct validity. Together, these factors suggest that Item 1 was heterogeneous relative to the remaining items and likely contributed to its deletion from the ERS-ACA-C.

In conclusion, several factors may have contributed to the heterogeneity of Item 1 in relation to the other content of the ERS-ACA-C. As a result, Chinese art students may have been less willing to accept this item when completing the scale, which in turn led to its low correlation with the total ERS-ACA-C score and ultimately to its deletion.

Due to the fact that, at present, the only standardized research instrument available in China for assessing emotion regulation related to artistic creation among university students majoring in art and design is the SERAMS—which we previously translated (DeVellis, 2016; Zumbo, 1999)—we adopted it to evaluate the criterion-related validity of the ERS-ACA-C. The SERAMS is the Chinese translation of the SERATS, which was developed by Haeyen et al. (2018) as a valid instrument for assessing the effects of art therapy in individuals with personality disorders. Its nine items cover respondents’ emotional awareness, expression, and regulation (i.e., emotional release, harmonization, or maintenance) following artistic creation (Haeyen et al., 2018; Haeyen and Noorthoorn, 2021). In the present study, the Pearson correlation coefficient between the total scores of the ERS-ACA-C and SERAMS was r = 0.721 (*p* < 0.001), indicating good criterion-related validity of the ERS-ACA-C. It also suggests that the two scales measure highly related but not identical constructs (Cohen, 2013). In terms of application, the ERS-ACA-C primarily focuses on capturing the immediate emotion regulation strategies employed by Chinese art and design university students during the process of artistic creation, whereas the SERAMS is more concerned with evaluating the outcomes of self-expression and emotional integration after the completion of artistic activities, placing greater emphasis on the end effects of emotion regulation.

Haeyen and Noorthoorn (2021) found that, when assessing undergraduate students majoring in art therapy, the three factors of the ERS-ACA (Avoidance Strategies, Approach Strategies, and Self-Development Strategies) were positively correlated with the total score of the SERATS, with Pearson correlation coefficients of *r* = 0.57, 0.81, and 0.62, respectively (*p* < 0.001). In the present study, the three factors of the ERS-ACA-C were also positively correlated with the total score of the SERAMS, but with slightly lower Pearson correlation coefficients of *r* = 0.556, 0.559, and 0.518, respectively (*p* < 0.001). These differences in criterion-related validity between the two studies may be attributed to several factors.

First, the two studies differ in terms of their backgrounds, sample, and application contexts. The sample in Haeyen’s study comprised undergraduate students majoring in art therapy in the Netherlands, whose creative activities were typically embedded within actual therapeutic and emotional regulation processes—that is, they would prefer to achieve emotional regulation through the means of artistic creation. In this case, they might be more sensitive to narratives that include methods and strategies. So, their approach strategies (such as acceptance, reappraisal, and problem solving) had a high correlation with SERATS (*r* = 0.81). By contrast, the sample used in the present study consisted of college students in China majoring in art and design, who were more often involved in academically oriented, technically demanding, and task-driven creative activities. Their creative processes were frequently shaped by classroom instruction, evaluations, or project constraints. That is, their emotion regulation was intended to serve the goal of achieving better outcomes in artistic production. In other words, unlike the Netherlands’ sample, their artistic creation is the goal rather than the means. In this context, their approach strategy factor had a relatively low correlation with SERAMS (*r* = 0.559).

Second, sample size and heterogeneity of the study population can also influence correlation coefficients. The sample in Haeyen’s study was small (*n* = 53), whereas the present study employed a much larger sample (*n* = 338). Correlation coefficients in small-sample studies tend to exhibit greater variability, which may result in coefficients that are either inflated or deflated relative to the actual correlation (Schönbrodt and Perugini, 2013). Moreover, the sample in Haeyen’s study was homogeneous—composed solely of undergraduate students in art therapy—which enhanced construct alignment and response consistency, thereby elevating the observed correlations. In contrast, the present study involved a highly heterogeneous sample in terms of both academic background and educational level, including undergraduate and graduate students across five creative directions within art and design major (Table 1), which increased response variability and weakened the linkage between emotion regulation strategy and outcomes, which contributed to lower correlation coefficients (Streiner et al., 2024).

In addition, differences in cultural backgrounds may also indirectly affect the correlation coefficients. The distinct cultural orientations of the East and West may lead students to perceive certain items on the scale differently when filling it out, thereby influencing their respective correlations. Netherlands’ students, living in a Western cultural context that encourages the open expression of emotions and emphasizes individuality and self-exploration, are more inclined to endorse the descriptions in the self-development strategies factor—which emphasizes personal identity, individual growth, and personal meaning—in the ERS-ACA (Miyamoto and Ma, 2011), resulting in a higher correlation (*r* = 0.62) between their self-development strategies factor score and the overall SERATS score. In contrast, Chinese students, who live in an Eastern cultural environment that esteems restraint and moderation, place greater importance on collective rather than individual identity factors and tend to be more reserved in expressing personal emotions (Miyamoto and Ma, 2011). They may exhibit a relatively lower level of identification with the items of the self-development strategies factor in the ERS-ACA-C, thus leading to a relatively weaker association (*r* = 0.518) with the SERAMS score.

The revised ERS-ACA-C has relatively good psychometric properties. The use of this scale will promote both the teaching and research work in Chinese art colleges. For example, in teaching art core courses, instructors can guide students to identify, reflect on, and regulate their emotions during the creative process, thereby helping them deepen their understanding of their own creative styles and psychological states. At the end of the course, instructors can also lead students in a creative journal review by completing the scale, which not only enhances individual awareness of emotion regulation but also serves as a basis for instructors to diagnose difficulties encountered in the creative process and to provide personalized guidance. Chinese educational researchers can employ the ERS-ACA-C to conduct longitudinal studies across different grade levels, tracking the evolution of emotion regulation strategies among students from various grades during their school years, and exploring the relationships among teaching, personal growth, and emotion regulation ability, thereby providing empirical evidence for curriculum reform and individualized training pathways. Psychological educators in art colleges can, based on the data from the ERS-ACA-C, develop psychological support programs for art college students, effectively enhancing their psychological resilience and empathy. Furthermore, the ERS-ACA-C can also serve as a tool for cross-disciplinary comparative research, allowing for the comparison of emotion regulation patterns in the creative processes among college students in different artistic fields—such as fine arts, design, drama, and music—thus providing a basis for devising specialized psychological intervention strategies.

## 5 Strengths

To our knowledge, ERS-ACA-C is the first tool to assess the emotion regulation strategies of Chinese college students majoring in art and design during their artistic creative activities. The sample size of this study meets the requirements for cross-cultural adaptation and validation of the scale (Costello and Osborne, 2005; Guillemin et al., 1993). The objectives of the study and the inclusion and exclusion criteria for subjects were clearly defined. The ERS-ACA-C has relatively good reliability and validity, and its structure has been confirmed by CFA. These factors represent the strengths of the study.

## 6 Limitations

Although this study has some strengths, it has a few limitations. The sample for this study was drawn from a specific group, which may limit the generalizability of the findings. The sample used to develop the ERS-ACA was drawn from across the UK and is more broadly representative. China is vast and populous. Art and design university students differ regarding socio-economic background, institutional resources, and regional cultural influences. The study’s sample size is relatively small, and all participants are from colleges in eastern provinces of China, which may not fully reflect the diversity of art and design students in China. Future studies should include multi-center, larger samples to enhance representativeness. Moreover, the current sample was primarily drawn from economically developed urban universities, which may not capture the full range of educational experiences and cultural backgrounds found in less-developed or rural regions of China. Students from such areas may be influenced by different institutional norms and community expectations, which could shape their emotion regulation strategies in unique ways. Future studies should therefore aim to include participants from a broader geographical and institutional spectrum, including rural colleges, small-town academies, and non-elite institutions. This would provide a more balanced and ecologically valid understanding of how emotional regulation manifests across diverse settings.

Second, item 1 did not receive an appropriate response in the Chinese research sample and was ultimately deleted, resulting in a slight difference in the structure of ERS-ACA-C compared to ERS-ACA. Influenced by traditional culture, Chinese people believe that overly absolute expressions of emotions can disrupt group harmony and status hierarchy, thus strictly regulating emotional expression (Bond, 1993). ERS-ACA is rooted in Western culture, so its content aligns with the emotional expression characteristics of Westerners. Although we strictly followed the scale revision standards, and ERS-ACA-C also has good psychometric properties, our transplantation work cannot fully match the emotional expression of Chinese people. Therefore, in the future, we must design research scales specifically for Chinese people from scratch and develop research tools that align with Chinese emotional expression, only in this way can we fully capture the subtle emotional expression strategies of Chinese students. In addition, the potential influence of language nuance and translation fidelity on item interpretation deserves further consideration. Certain concepts embedded in the original ERS-ACA items may not have fully transferred culturally or linguistically despite careful translation. Future scale development efforts should engage in more rigorous cross-cultural adaptation procedures, including cognitive interviews and pilot testing across subpopulations, to ensure conceptual and semantic equivalence.

Third, the exclusive reliance on self-report measures poses significant methodological limitations, particularly in cross-cultural research contexts such as this one. Self-report instruments, while commonly used in psychological studies, are inherently susceptible to social desirability bias, memory distortions, and culturally influenced response styles. In Chinese educational environments—where emotional restraint, collectivism, and the maintenance of group harmony are culturally emphasized—students may consciously or unconsciously underreport internal emotional experiences such as suppression, or overreport culturally valued strategies like cognitive reappraisal. These factors could distort the data and obscure the full range of emotional regulation strategies employed by participants. Moreover, the absence of supplementary data sources, such as behavioral tasks, informant ratings, or observational assessments, limits the ability to validate whether participants’ reported behaviors align with actual emotional practices—especially in studio-based or performative educational settings. To enhance the validity and ecological relevance of future findings, researchers are encouraged to adopt a multi-method approach, incorporating self-report data alongside qualitative interviews, diary methods, ecological momentary assessments, or structured behavioral observations in naturalistic artistic activities. Such triangulation would enrich the data, mitigate introspective biases, and offer a more comprehensive understanding of emotion regulation in creative educational contexts.

Fourth, the subjects for this study were drawn from five fine art school majors: Environmental Art Design, Graphic Design, Public Art Design, Fine Art Education, and Painting. However, the adaptation of ERS-ACA-C ignores potential differences between the aforementioned art subdisciplines, which may have resulted in the loss of information unique to a particular subdiscipline. Different artistic practices may trigger different emotion regulation strategies. Treating these sub-disciplines as homogeneous may cause research to overlook the unique patterns of different sub-disciplines. Future research should conduct subgroup analyses to explore the specific responses of each sub-discipline. Notably, students enrolled in performing arts programs such as theater, dance, or music may exhibit distinct emotional processes compared to those in visual or design-oriented disciplines. Their training often involves embodied expression and live interaction, which may cultivate different emotional awareness and regulation patterns. The current study does not encompass such artistic domains. Expanding validation efforts to include performing arts and other creative domains would enhance the comprehensiveness and applicability of the ERS-ACA-C across the spectrum of artistic education.

Fifth, the exclusive reliance on self-report measures introduces methodological challenges that are particularly salient in cross-cultural research. While self-report instruments are widely used in psychological research, they are vulnerable to social desirability bias, memory inaccuracies, and culturally influenced response patterns. In Chinese educational contexts, where emotional restraint, collectivism, and group harmony are culturally emphasized, students may hesitate to disclose internal emotional experiences such as suppression, or may amplify socially valued strategies like cognitive reappraisal. This could lead to distorted reporting and obscure the true variability of emotional regulation styles. Moreover, the study lacks complementary methodological strategies such as behavioral observations, informant reports, or diary-based records, which limits the ability to verify whether reported behaviors correspond to actual regulatory practices, particularly in studio-based or performance settings. Future research should adopt a multi-method approach by integrating self-reports with qualitative interviews, ecological momentary assessments, or structured observations during artistic activities, thereby enriching the data, enhancing validity, and mitigating the limitations of introspective self-reporting.

## 7 Conclusion

We verified the ERS-ACA-C using Chinese college students majoring in art and design as the research subjects. ERS-ACA-C has shown relatively good psychometric properties, with a general factor and three factors comprising 17 items. It can be used to assess the emotion regulation strategies of Chinese college students majoring in art and design in their artistic creative activities.

## Data Availability

The datasets presented in this article are not readily available because they are part of a continuing study and access is currently restricted. Requests to access the datasets should be directed to the corresponding author upon reasonable request after the completion of the entire study.
